# Inhibition of CDCP1 by 8‐isopentenylnaringenin synergizes with EGFR inhibitors in lung cancer treatment

**DOI:** 10.1002/1878-0261.13429

**Published:** 2023-04-20

**Authors:** Sze‐Ching Wong, Chun‐Chieh Yeh, Xun‐Yu Zhang, Chih‐Ying Hsieh, Chia‐Chien Lo, Ting‐Ting Kuo, Ching‐Chan Lin, Chih‐Hua Chao, Jing‐Pei Liu, Ling‐Chu Chang, Lu‐Hai Wang, Yuh‐Pyng Sher

**Affiliations:** ^1^ Graduate Institute of Biomedical Sciences China Medical University Taichung Taiwan; ^2^ Chinese Medicine Research Center China Medical University Taichung Taiwan; ^3^ Research Center for Chinese Herbal Medicine China Medical University Taichung Taiwan; ^4^ Department of Medicine, School of Medicine China Medical University Taichung Taiwan; ^5^ Department of Surgery, Organ Transplantation Center China Medical University Hospital Taichung Taiwan; ^6^ Center for Molecular Medicine China Medical University Hospital Taichung Taiwan; ^7^ Division of Hematology and Oncology China Medical University Hospital Taichung Taiwan; ^8^ School of Pharmacy China Medical University Taichung Taiwan; ^9^ Chinese Medicine Research and Development Center China Medical University Hospital Taichung Taiwan; ^10^ Graduate Institute of Integrated Medicine China Medical University Taichung Taiwan

**Keywords:** 8‐isopentenylnaringenin, EGFR TKI acquired resistance, lung adenocarcinoma, necrosis, synergistic effects

## Abstract

CUB domain‐containing protein 1 (CDCP1) contributes to epidermal growth factor receptor (EGFR) tyrosine kinase inhibitor (TKI) resistance by regulating EGFR signaling pathways and is a potential target in lung cancer treatment. This study aims to identify a CDCP1 reducer that synergistically improves TKI treatment. Utilizing a high‐throughput drug screening system, a phytoestrogen 8‐isopentenylnaringenin (8PN) was identified. Upon 8PN treatment, CDCP1 protein levels and malignant features were reduced. 8PN exposure caused the accumulation of lung cancer cells in G0/G1 phase and increased the proportion of senescent cells. In EGFR TKI‐resistant lung cancer cells, the combination of 8PN and TKI synergistically reduced cell malignance, inhibited downstream EGFR pathway signaling, and exerted additive effects on cell death. Moreover, combination therapy effectively reduced tumor growth and enhanced tumor necrosis in tumor xenograft mice models. Mechanistically, 8PN increased interleukin (IL)6 and IL8 expression, induced neutrophil infiltration, and enhanced neutrophil‐mediated cytotoxicity to attenuate lung cancer cell growth. In conclusion, 8PN enhances the anticancer efficacy of EGFR TKI on lung cancer and triggers neutrophil‐dependent necrosis, highlighting the potential to overcome TKI resistance in lung cancer patients who have *EGFR* mutation.

## Graphical abstract

CUB domain‐containing protein 1 (CDCP1) contributes to epidermal growth factor receptor (EGFR) tyrosine kinase inhibitor (TKI) resistance in lung cancer treatment. 8‐isopentenylnaringenin (8PN) was identified as a CDCP1 reducer to suppress CDCP1 proteins via lysosomal degradation and reduced lung cancer progression. The combination of 8PN and EGFR TKIs showed a synergistic anticancer effect. Mechanically, 8PN increases interleukin (IL)6 and IL8 expressions to recruit neutrophils, leading to reactive oxygen species production for tumor necrosis.

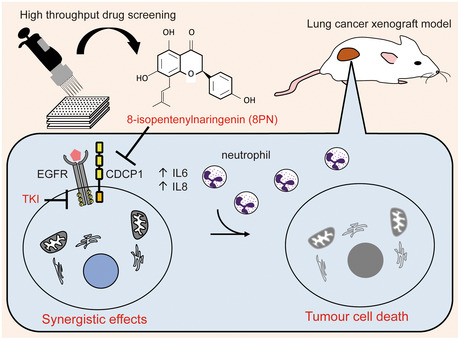

**Abbreviations**
8PN: 8‐isopentenylnaringenin
CDCP1: CUB domain‐containing protein 1
CI: combination index
DELFIA: dissociation‐enhanced lanthanide fluorescence immunoassay
EGFR: epidermal growth factor receptor
ER: estrogen receptor
MPO: myeloperoxidase
NAC: *N*‐acetyl cysteine
NSCLC: non‐small‐cell lung cancer
ROS: reactive oxygen species
shRNA: short hairpin RNA
TKI: tyrosine kinase inhibitor

## 1 Introduction

Non‐small‐cell lung cancer (NSCLC) is the most common type of lung cancer, and many NSCLC patients are diagnosed at advanced stages, two‐thirds of which harbor epidermal growth factor receptor (EGFR) mutations [1]. EGFR tyrosine kinase inhibitors (TKIs) have been widely applied in the first‐line treatment of NSCLC patients [2]. However, acquired resistance and adverse effects are commonly reported [3]. There is thus a need for alternative therapeutic solutions to enhance the efficacy of EGFR TKIs and reduce the occurrence of TKI resistance in lung cancer treatment.

CUB domain‐containing protein 1 (CDCP1) is a type I transmembrane glycoprotein [4]. Two forms of CDCP1, full‐length and truncated, can be found on the cell surface. Elevated cleaved CDCP1 proteins were detected in brain‐metastatic lung cancer and breast cancer sublines compared with their parental cells [5]. Numerous reports show that upregulated CDCP1 is commonly found in cancer patients, many of whom show poor overall survival probability [4]. Given that CDCP1 regulates several signaling pathways for tumor malignancy, it could be a potential target for cancer treatment.

It has demonstrated that CDCP1 may be a key mediator of EGFR TKI resistance via its direct interaction with EGFR [4]. Hence, the development of effective CDCP1‐targeted therapy may help to ameliorate resistance to EGFR TKI treatment. To develop potential CDCP1 inhibitors, we performed high‐throughput drug screening with a cell‐based platform and identified a prenylated flavonoid, 8‐isopentenylnaringenin (8PN), as a potential agent to suppress CDCP1 expression and influence lung cancer progression. Here we describe the effects of 8PN on lung cancer cells, most notably its synergism with EGFR TKIs in TKI‐resistant cells. Our studies reveal that 8PN has the potential for further development as an adjuvant for the treatment of lung cancer patients with EGFR mutations.

## 2 Materials and methods

### 2.1 High‐throughput screening of small molecules using the DELFIA assay

The dissociation‐enhanced lanthanide fluorescence immunoassay (DELFIA), a time‐resolved assay with high sensitivity and a wide dynamic range, was used in high‐throughput screening to identify potential CDCP1 inhibitors. The high‐throughput screening was performed in ChemBank at the High‐Throughput Screening Resource Center, Academia Sinica, using a representative compound library of 160 000 molecules. The DELFIA assay was conducted according to the manufacturer's protocol. In brief, human lung adenocarcinoma F4 cells (RRID:CVCL_A5BR) were infected with pLNCX‐HA‐dsCDCP1, a retroviral plasmid designed to express N‐terminal HA‐tagged CDCP1 protein from the pLNCX vector. The stable cancer cell population continuously expressing HA‐CDCP1 fusion proteins on cell membranes was selected by G418. Cells (400 cells per well) were subsequently cultured for 2 days in 1536‐well plates, which contained 6.25 μm small molecule per well. After drug treatment, cells were stained with Eu‐N1‐anti‐HA antibody (at 1 : 200 dilution, Cat#AD0054; Perkin Elmer, Waltham, MA, USA) in DELFIA® Assay Buffer (Cat#1244‐111; Perkin Elmer). After five cycles of washing using DELFIA® Wash Buffer (Cat#1244‐114; Perkin Elmer) and adding DELFIA® Enhancement solution (Cat#1244‐105; Perkin Elmer) for 30‐min incubation, time‐resolved fluorescence was determined by a reader at em = 615 nm and ex = 340 nm. The CDCP1 expression was calculated by comparing fluorescence signals in drug‐treated groups with the vehicle control group. Cancer cell viability after drug treatment was also measured using a CellTiter‐Glo Luminescence Cell Viability assay (Promega, Madison, WI, USA).

### 2.2 Cell culture and reagents

Human lung adenocarcinoma F4 cells (RRID:CVCL_A5BR) were gifted from National Taiwan University, and the subline of F4 cells metastasized to mice brain (Bm7 (RRID:CVCL_A5BS)) were established as previously described [5]. Human lung cancer cell lines [H1975 (RRID: CVCL_1511) and H1650 (RRID:CVCL_1483)] were gifted by J.‐Y. Shih (National Taiwan University), and a mouse lung cancer cell line (TC1) were gifted by T. C. Wu (Johns Hopkins University). Cells were maintained in RPMI‐1640 medium (Thermo Fisher Scientific, Waltham, MA, USA) supplemented with 10% FBS and 1% penicillin/streptomycin solution (Gibco). Cells were free of mycoplasma contamination and have been authenticated in 2020 by short tandem repeat genotyping.

MG132, chloroquine (CQ), afatinib, and *N*‐acetyl cysteine (NAC) were purchased from Sigma‐Aldrich (St. Louis, MO, USA). 8PN and AZD9291 were purchased from Enzo Life Science (Farmingdale, NY, USA), and MedChemExpress (Monmouth Junction, NJ, USA), respectively. Afatinib and AZD9291 are EGFR TKIs. Inhibitors for different cell death pathways were used, including ferrostatin‐1 (#17729; Cayman Chemical), deferoxamine (#D9533; Sigma‐Aldrich), pan‐caspase inhibitor Z‐VAD‐FMK (#14463; Cayman), necrostatin‐1 (#11658; Cayman), necrosulfonamide (#5025; Tocris Bioscience, Bristol, UK), and inflammasome inhibitors MCC‐950 (#17510; Cayman) and VX765 (#28825; Cayman). Anti‐IL6 receptor antibody (Tocilizumab; Actemra) was purchased from Roche Diagnostics (Indianapolis, IN, USA).

### 2.3 Immunoblotting and antibodies

Cells treated with 8PN and/or EGFR TKI (afatinib or AZD9291) were lysed in NETN lysis buffer (150 mm NaCl, 1 mm EDTA pH 8.0, 20 mm Tris–HCl pH 8.0, 0.5% NP40) after 24 h. Immunoblotting was conducted as previously described [6], except that we used total of 10% sodium dodecyl sulfate–polyacrylamide gels for protein separation. After 1 h of blocking, primary antibodies were used against the following targets: CDCP1 (ab1377; Abcam, Cambridge, MA, USA), retinoblastoma protein (RB; #2947; Cell Signaling Technology, Danvers, MA, USA), phosphorylated RB (#8516; Cell Signaling Technology), cyclin E1 (ab33911; Abcam), cyclin E2 (ab40890; Abcam), EGFR (sc‐373746; Santa Cruz, Dallas, Texas, USA), phosphorylated EGFR (p‐EGFR; ab1377 and ab32894; Abcam), phosphorylated ERK (p‐ERK; #9101; Cell Signaling Technology), ERK (#9102; Cell Signaling Technology), and caspase 3 (#9661s; Cell Signaling Technology, or ab1899; Millipore, Billerica, MA, USA). GAPDH (10494‐1‐AP; ProteinTech, Rosemont, IL, USA), and EF1α (#05‐235; Millipore) were used as internal controls. Blotted proteins were detected using an enhanced chemiluminescence system (Millipore) with the BioSpectrum Imaging System (UVP, Upland, CA, USA).

### 2.4 Time‐lapse migration assay

Cancer cells were cultured in 24‐well plates overnight and then incubated in serum‐free culture medium (for Bm7, TC1, and H1650) or 1% FBS (for H1975) containing various doses of 8PN. The time‐lapse measurements for migration were detected by using Cytation 5 (BioTek Instruments, Winooski, VT, USA) and performed for 17 h (TC1, H1975, and H1650) or 7 h (Bm7) with a time interval of 30 min (TC1, H1975, and H1650) or 20 min (Bm7) using a 4× objective. Thirty cells were tracked from each group. The accumulated distance was measured by tracking each cell using the Track Point function of imagej software (version 1.52a; Wayne Rasband, National Institutes of Health, Bethesda, MD, USA).

### 2.5 Cell viability assay

Cell viability was analyzed using Cell Counting Kit‐8 (CCK‐8; Dojindo Laboratories, Kumamoto, Japan). Briefly, cells were cultured with drugs for 72 h in 100 μL medium in plates. Subsequently, 10 μL of CCK‐8 solution was added to each well and incubated at 37 °C for 4 h, and the absorbance was measured at 450 nm using a microplate reader (Bio‐Rad, Hercules, CA, USA). In crystal violet assay, cultured cells were fixed with 10% formaldehyde and stained with 0.01% crystal violet for 1 h. Cell grown area was calculated using imagej software (Wayne Rasband, National Institutes of Health, Bethesda, MD, USA).

### 2.6 Colony formation assay

A total of 100 cells from each group were cultured in a 6‐well plate for 7–14 days. The cells were then fixed with 10% formaldehyde and subsequently stained with 0.01% crystal violet stain for 10 min. Following rinsing with PBS, the colonies formed in each well were counted using Cytation 5 and imagej. The plating efficiency was calculated as a percentage by dividing the number of colonies formed by a number of cells seeded.

### 2.7 Sphere formation assay

The sphere formation assay was performed as previously described [7]. Briefly, 5 × 10^3^ lung cancer cells were cultured on 1% agarose‐coated 6‐cm dishes in DME/F12 (Bm7) or RPMI‐1640 (TC1 and H1650) media supplemented with N‐2 (Life Technologies), 10 ng·mL^−1^ human recombinant fibroblast growth factor basic, 10 ng·mL^−1^ epidermal growth factor, and 1% penicillin/streptomycin (Life Technologies). The sphere number was determined within 1–2 weeks.

### 2.8 Cell cycle assay

The position in the cell cycle was detected by flow cytometry. After 24‐h culture in serum‐free media, cells were treated with different concentrations of 8PN and/or EGFR TKI (afatinib or AZD9291). Then, the cells were harvested and fixed in 70% ethanol at 4 °C. After fixation, the cells were centrifuged at 300 ***g*** for 5 min to remove the ethanol. Following PBS washings, cells were incubated with 20 μg·mL^−1^ of RNase A at 37 °C for 15 min and stained with 20 μg·mL^−1^ propidium iodide. Cell cycle distribution was analyzed using a FACSVerse™ flow cytometer (BD Biosciences, Franklin Lakes, NJ, USA). Cell distribution in different stages of the cell cycle was analyzed using bd facsuite software (BD Biosciences).

### 2.9 Senescence‐associated β‐galactosidase assay

This assay was performed as previously described [8]. Briefly, cells were plated in a 24‐well plate and treated with different doses of 8PN for 4 days (TC1) or 6 days (Bm7 and H1650). Cells were fixed and stained following the manufacturer's protocol (Senescence β‐Galactosidase Staining Kit; Cell Signaling Technology #9860). The staining images were captured using Cytation 5. Three random images were recorded, and cells were counted by imagej software.

### 2.10 Synergy assay

The therapeutic effect of a two‐drug combination was assessed by cell viability assays, and the combination index (CI) was calculated by the calcusyn program (Biosoft, Cambridge, UK), as previously described [9]. A CI < 1 is indicative of a synergistic effect.

### 2.11 CDCP1 knockdown

Lentiviral short hairpin RNAs (shRNAs) targeting CDCP1 (clone #1: TRCN0000136536; clone #2: TRCN0000134829) were obtained from the National RNAi Core Facility located at the Institute of Molecular Biology, Genomic Research Center, Academia Sinica.

### 2.12 Tumor xenograft animal models

The lung tumor xenograft mouse model was prepared as previously described [10]. Animal experiments were performed in accordance with the guidelines and regulations at China Medical University, Taiwan, and were approved by the Institutional Animal Care and Use Committee (IACUC) of China Medical University (animal protocol no. 2018‐142). Mice were housed in specific pathogen‐free rooms as five mice in one cage at room temperature (20–23 °C), 40–60% relative humidity, and under a 12‐h light–dark cycle. They were free to access to food and water. SCID male mice were purchased from the National Laboratory Animal Center (Taipei, Taiwan). Luciferase‐expressing H1650 cells (1 × 10^6^ cells) were subcutaneously implanted in the mice at 6 weeks of age on both sides of the back. Ten days later, mice were randomized into vehicle or treatment groups and treated with 8PN, afatinib, or the combination by different routes every 5 days per week for 3 weeks. Briefly, 8PN, dissolved in 30% hydroxypropyl‐β‐cyclodextrin (Sigma) [11], was subcutaneously administrated to the mice at a dose of 10 mg·kg^−1^. Afatinib was suspended in 10% DMSO, 40% PEG300, 5% Tween‐80, and 45% saline, and was orally administrated at a dose of 5 mg·kg^−1^.

To examine the synergistic effects of 8PN and AZD9291, an H1975 xenograft mouse model was used. H1975 cells (0.75 × 10^6^) with stable luciferase expression were mixed with Matrigel and subcutaneously injected into 6–8‐week‐old male SCID mice on both sides of the back. Ten days later, they were randomly divided into four treatment groups: mock control (vehicle, 30% hydroxypropyl‐β‐cyclodextrin), 8PN in vehicle solution, AZD9291 (AZD; dissolved in 10% DMSO, 40% PEG300, 5% Tween‐80, and 45% saline), and combination of 8PN and AZD. 8PN (10 mg·kg^−1^) was subcutaneously injected daily, and AZD (0.1 mg·kg^−1^) was given orally daily for 4 weeks.

The size of H1650 and H1975 tumors was detected with an IVIS spectrum imaging system (Xenogen, Hopkinton, MA, USA) once a week. Meanwhile, body weight was recorded. Tumor sample preparation and histology from tumor‐bearing animals were fixed in 4% neutral‐buffered formalin and submitted to RAPID Science Co. Ltd. (Taichung, Taiwan) to be cut into 4‐μm‐thick slices and stained with hematoxylin & eosin (H&E). At least three random fields from HE‐stained sections per mouse were taken using a Lecia microscope (Deerfield, IL, USA), and necrotic areas of tumors were measured by imagej software. The necrotic area was defined as the death of cells in the disappearance of a nucleus in the tumors.

### 2.13 Quantitative reverse transcription PCR

Human lung cancer cells, treated with 8PN and/or EGFR TKI, were harvested 24 h postexposure. Total RNA was isolated by Trizol reagent (Invitrogen, Carlsbad, CA, USA) according to the manufacturer's protocol. Five microgram total RNA was reverse transcribed into cDNA using SuperScript™ III Reverse Transcriptase (Invitrogen) according to the manufacturer's protocol. cDNA was subsequently eluted in nuclease‐free water. Subsequently, quantitative PCR was performed using KAPA SYBR® Master Mix. Reactions were run on a LightCycler 480 real‐time PCR system (Roche) using the following conditions: preincubation at 94 °C 10 min, followed by 50 cycles of 94 °C for 15 s, 60 °C for 1 min, and 72 °C for 1 min. Primer sets for IL6 RNA: forward primer, 5′‐TTCGGTCCAGTTGCCTTCTC‐3′; reverse primer, 5′‐TCTTCTCCTGGGGGTACTGG‐3′. Primer sets for IL8 RNA: forward primer, 5′‐CAGAGACAGCAGAGCACACA‐3′; reverse primer, 5′‐ACAGTGAGATGGTTCCTTCCG‐3′.

### 2.14 Immunohistochemical staining

Immunohistochemistry was carried out as previously described [8]. Briefly, tissue sections were blocked in 2.5% horse sera and incubated with primary antibody against myeloperoxidase (MPO; #A0398; Agilent DAKO, Santa Clara, CA, USA) overnight at 4 °C. Counterstaining was performed with hematoxylin. MPO‐positive cells were assessed by imagej software from nine random high‐power fields.

### 2.15 Neutrophil and H1650 co‐culture assay

Human blood was collected from a healthy individual in EDTA tubes following informed and written consent. Human neutrophils were isolated from peripheral blood by density gradient centrifugation. The study methodologies conformed to the standards set by the Declaration of Helsinki and were approved by the Institutional Review Board of China Medical University Hospital (CMUH111‐REC3‐153 (AR‐1)). In brief, neutrophils were separated from peripheral blood mononuclear cells using Ficoll‐Paque™ PREMIUM (1.084 g·mL^−1^; GE Healthcare, Chicago, IL, USA) and were sedimented using a 3% dextran/NaCl solution (Dextran sulfate sodium, M.W. ~ 500 000; VWR Life Science, Radnor, PA, USA) and Hank's balanced salt solution (Gibco). Red blood cells were lysed by a custom buffer (1.5 m NH_4_Cl, 100 mm NaHCO_3_, 10 mm disodium EDTA, pH 7.4) for 5 min at room temperature, followed by centrifugation and resuspension of neutrophils in RPMI supplemented with 10% heat‐inactivated FBS. Neutrophils were subsequently pretreated with 8PN for 24 h before the lung cancer cell co‐cultures. H1650 cancer cells were seeded (50 cells per well) in 12‐well culture plates. After 24 h, the medium was removed, and H1650 cells were incubated with 40 μm 8PN. After an additional 24 h, 8PN‐treated neutrophils and 8PN‐treated H1650 cells were continuously co‐cultured for 5 days, followed by colony formation assays.

### 2.16 Net reactive oxygen species measurement in human neutrophils

1 × 10^5^ neutrophils were cultured and treated with 8PN for 30 min. Upon incubation, cells were washed with PBS and incubated with 10 μm of 2′,7′‐dichlorodihydrofluorescein diacetate (DCFH‐DA) for 30 min. After PBS washes, reactive oxygen species (ROS) production as indicated by DCFH‐DA was measured by a flow cytometer (FACS Verse; Becton Dickinson, Franklin Lakes, NJ, USA). Ethical approval was approved by the hospital's institutional review board (IRB) of China Medical University Hospital.

### 2.17 Statistical analysis

Statistical analysis was conducted using graphpad prism 5 software (GraphPad Software, Inc., San Diego, CL, USA). Unpaired *t*‐tests were used to examine colony formation, anchorage‐independent cell growth, migration, and cell senescence. One‐way ANOVA was used to assess the necrotic area and MPO‐positive lung cancer cells. Statistical significance was determined by *P* < 0.05.

## 3 Results

### 3.1 8PN was identified as a CDCP1 inhibitor by high‐throughput screening in a lung cancer cell‐based system

**Fig. 1:**
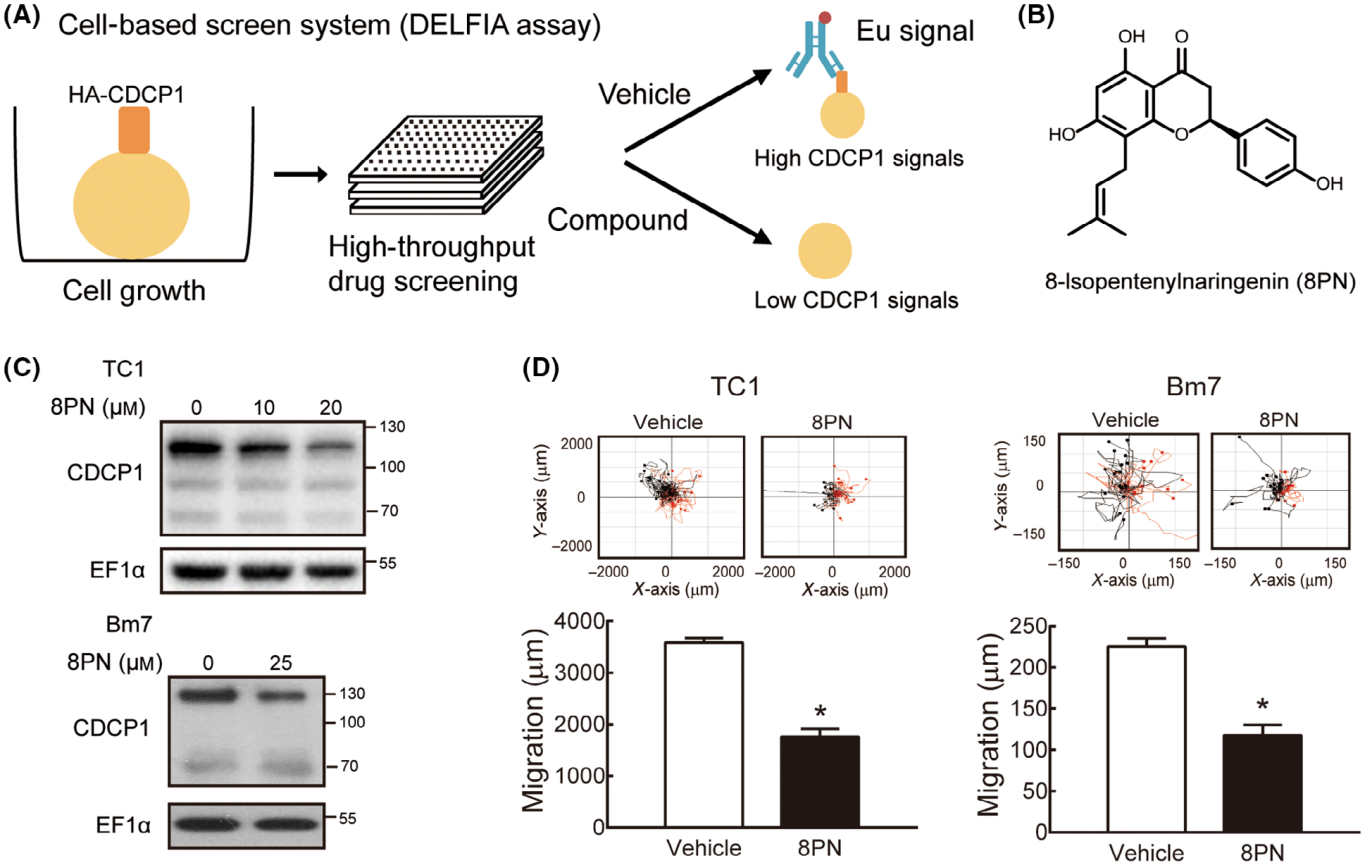
8‐Isopentenylnaringenin is identified from a compound library by screening its capacity for CDCP1 reduction and has antimigration effects on lung cancer cells. (A) Schematic design of screening CDCP1 inhibitors. (B) The top compound, 8PN, was identified by our cell‐based screen. The structure was drawn using chembiodraw ultra 14.0 (CambridgeSoft corporation, Cambridge, MA, USA). (C) Immunoblotting analysis of CDCP1 in TC1 and Bm7 lung cancer cells after 8PN treatment for 24 h. EF1α was the loading control. Similar results were detected at least three times. (D) Migration distance of lung cancer cells was measured by time‐lapse migration assays in TC1 cells with 20 μm 8PN and in Bm7 cells with 25 μm 8PN. Each colored dot and line represents one trace of an individual cell. Black and red lines represent movement in opposite directions. Statistical analyses were determined by the Student's *t*‐test. Data are shown as mean ± standard deviation. Experiments were performed in three replicates. *P < 0.05.

To identify the small compounds that reduce CDCP1 expression, we have established a cell‐based system with DELFIA for high‐throughput screening of a library of 160 000 compounds (Fig. 1A). Detected signals, representing the HA‐tagged CDCP1 levels on the surface of lung cancer cells, were compared between the treated samples and the untreated control group. Based on two independent cell‐based screening DELFIA assays, which showed no effect on cell viability at 6.25 μm, 8PN was identified as the top candidate to suppress CDCP1 levels on the surface of lung cancer cells (Fig. 1B). We confirmed that full‐length CDCP1 proteins were exclusively reduced, although a small portion of the cleaved CDCP1 was detected by treating mouse lung cancer TC1 cells and human lung cancer Bm7 cells with 8PN (Fig. 1C). Although CDCP1 cleavage is necessary for tumor aggressiveness [12], 8PN majorly reduced the full‐length CDCP1 proteins. Notably, CDCP1 mRNA levels were not reduced when cells were treated with 8PN (Fig. S1A). We found that 8PN reduced CDCP1 proteins, and proteasome inhibitor MG132 did not restore the 8PN‐mediated CDCP1 reduction (Fig. S1B). However, chloroquine (CQ) that blocks lysosomal degradation showed a rescue of CDCP1 proteins in Bm7 and H1650 cells with 8PN treatment (Fig. S1B), implicating 8PN‐induced CDCP1 protein suppression undergoes lysosomal degradation pathways.

Reducing CDCP1 protein expression has been reported to decrease cell migration ability [7]. Owing to the effects of 8PN on CDCP1 protein downregulation, we sought to examine whether 8PN could influence the migration ability of lung cancer cells using a time‐lapse migration assay. With 8PN treatment, the migration distance of the lung cancer cells was dramatically decreased compared with vehicle control in human and mouse lung cancer cell lines (Fig. 1D). These results demonstrate that 8PN reduces CDCP1 expression and impedes lung cancer cell migration.

### 3.2 8PN inhibits cell proliferation and stemness properties of lung cancer cells

**Fig. 2:**
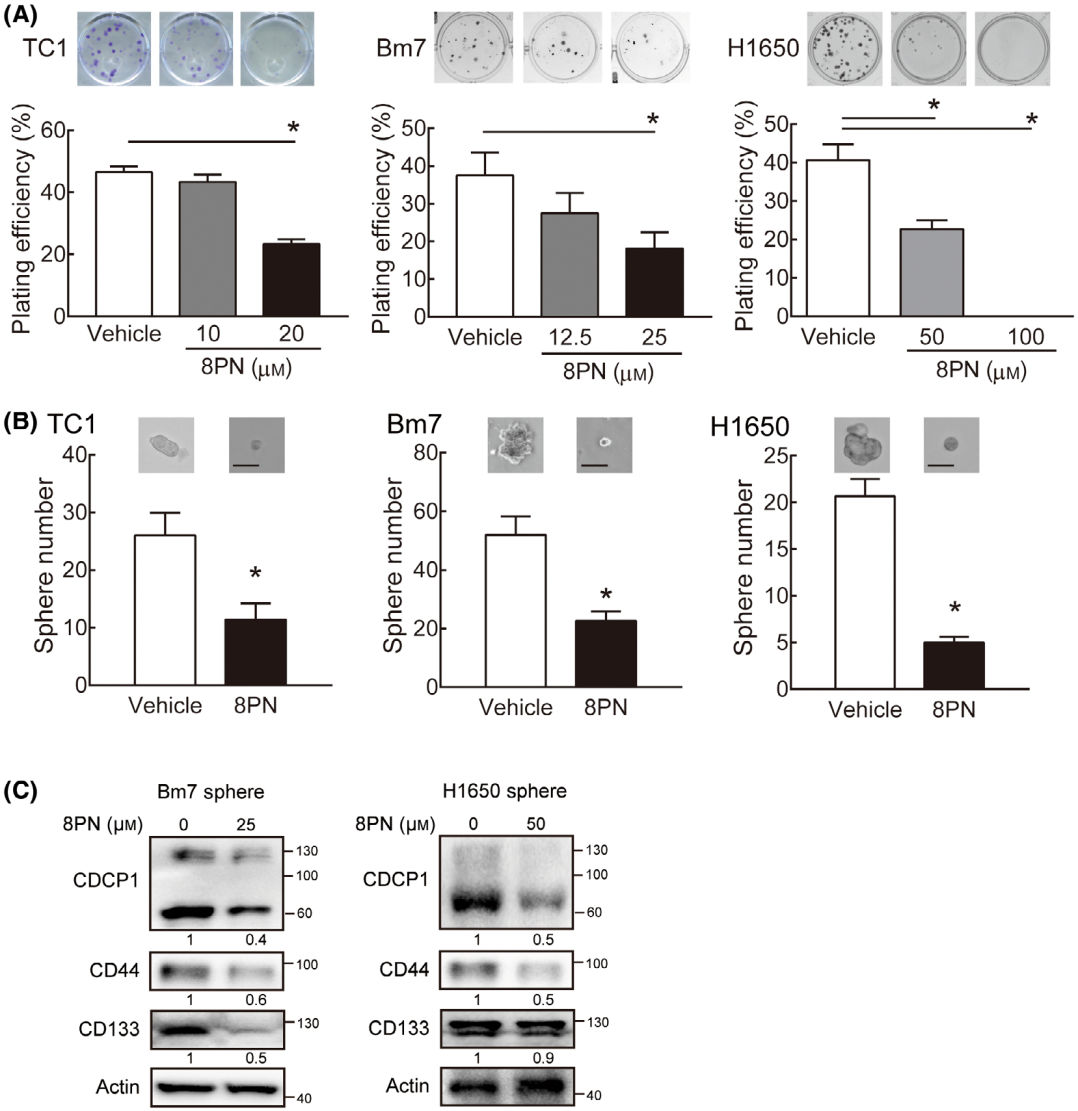
8‐Isopentenylnaringenin dose‐dependently suppresses lung cancer colony formation and sphere formation ability. (A) Colony formation was analyzed after 7 days of 8PN treatment in TC1, Bm7, and H1650 cells. Experiments were performed in three replicate wells. Statistical analyses were determined by the Student's *t*‐test. Data are shown as mean ± standard deviation. *P < 0.05. (B) The number of spheres was measured 12 days post‐treatment in TC1 cells with 20 μm 8PN, Bm7 cells with 25 μm 8PN, and H1650 cells with 12.5 μm 8PN (*n* = 3). Scale bar, 50 μm. Statistical analyses were determined by the Student's *t*‐test. Data are shown as mean ± standard deviation. *P < 0.05. (C) Immunoblotting analysis of CDCP1, CD44, and CD133 in Bm7 sphere cells with 25 μm 8PN and H1650 sphere cells with 50 μm 8PN for 9 days. Actin was the loading control. Experiments were repeated in three times.

Next, we investigated whether 8PN can reduce cell proliferation of lung cancer cells with different EGFR mutation status, including TC1 cells (EGFR wild‐type), Bm7 cells (EGFR wild‐type), and H1650 cells (EGFR exon 19 deletion and PTEN mutant). Regardless of the EGFR type, 8PN reduced the viability of lung cancer cells in a dose‐dependent manner after 3 days of treatment, with IC_50_ values ranging from 22 to 100 μm, whereas it had no cytotoxicity in mouse splenocytes (Fig. S1C). Similarly, 8PN influenced the clonogenic ability of lung cancer cells in a dose‐dependent manner (Fig. 2A). These data indicate that 8PN suppresses the cell viability and colony formation ability of lung cancer cells.

CUB domain‐containing protein 1 has been reported to contribute to anchorage‐independent cell growth to overcome anoikis for cancer progression [13]. Based on the observation that anchorage‐independent proliferation of tumor spheres is a hallmark of malignancy in cancer stem cells [14], next, we evaluated the effects of 8PN on cancer cells by measuring sphere formation. Sphere formation of our panel of lung cancer cells was significantly impaired by 8PN, compared with their respective vehicle controls (Fig. 2B). We also introduced lentiviral vector‐based shRNA to suppress CDCP1 expression in Bm7 cells and verified its impacts on sphere formation ability. CDCP1 protein abundance was reduced by shRNA, and sphere formation capacity was lower in the *CDCP1* knockdown group (Fig. S2A). Consistent with these findings, immunoblotting results verified that stem cell markers CD44 and CD133 proteins were significantly downregulated in 8PN‐treated Bm7 and H1650 cells compared with vehicle control (Fig. 2C). This reveals that 8PN abolishes the maintenance of cancer stem cell properties in lung cancer cells.

Together, our results revealed that 8PN reduced CDCP1 proteins and alleviate lung cancer growth. Given that blockage of the extracellular portion of cleaved CDCP1 stimulates cell apoptosis [12], we investigated whether 8PN activates caspase 3 following inducing intrinsic apoptosis via CDCP1 overexpression and suppression systems. We found that 8PN treatment markedly increased cleaved caspase 3 in the CDCP1 overexpression group compared with the vector group (Fig. S2B). In addition, 8PN‐induced cleaved caspase 3 was undetectable in CDCP1 knockdown cells compared with shVOID control cells (Fig. S2B), suggesting that 8PN triggers cell death through CDCP1 reduction.

### 3.3 8PN induces lung cancer cell senescence

**Fig. 3:**
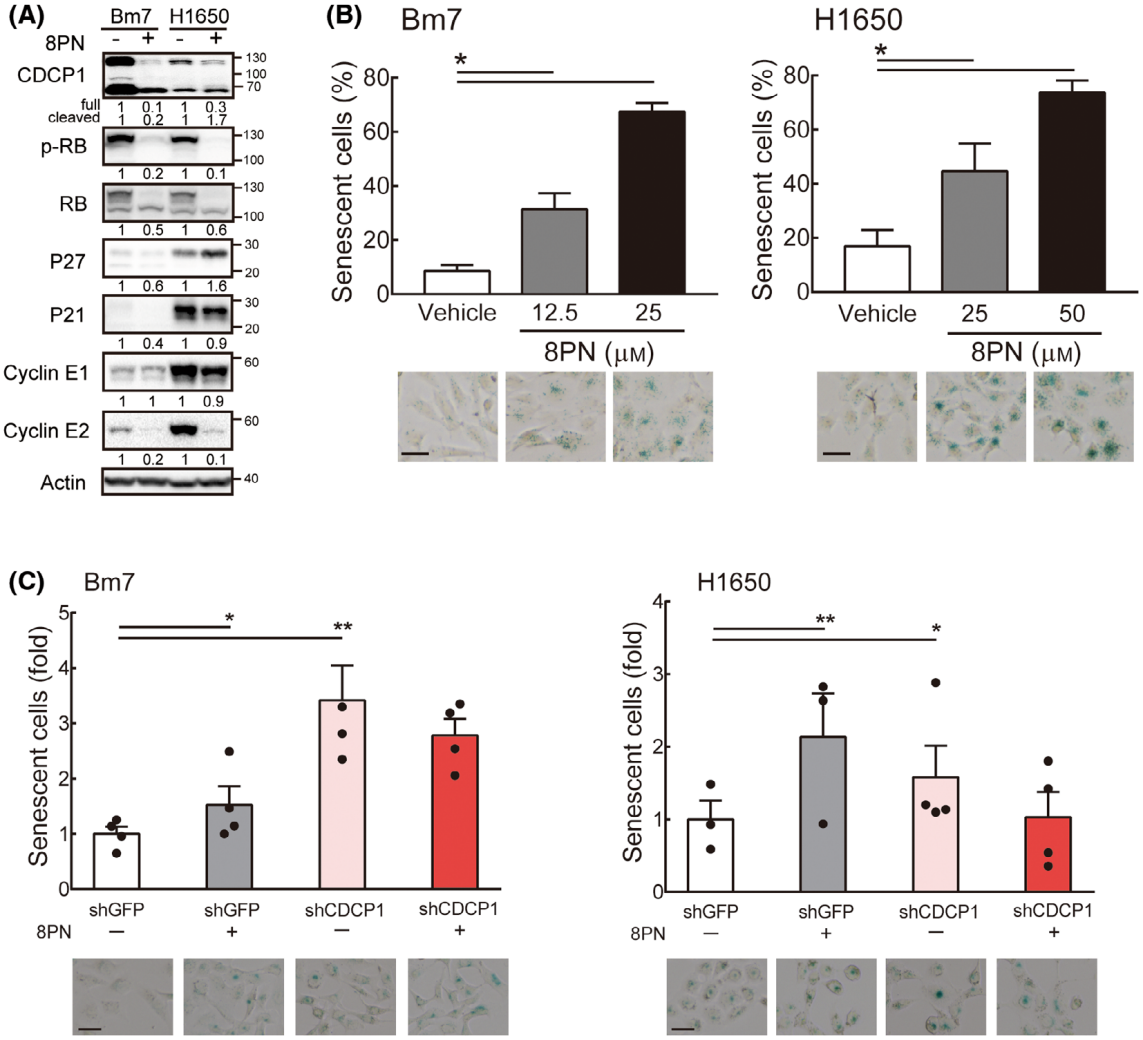
8‐Isopentenylnaringenin induces cell cycle arrest in G0/G1 phase and senescence. (A) Immunoblotting analysis of cell cycle proteins in Bm7 cells with 25 μm 8PN and H1650 cells with 50 μm 8PN for 48 h. Actin was the loading control. (B) β‐Galactosidase staining in lung cancer cells after treatment with the indicated dose of 8PN for 6 days. The number of senescent cells was quantified using the imagej software. Representative images shown in the lower panel. Scale bar, 40 μm. Statistical analyses were determined by the Student's *t*‐test. (C) β‐Galactosidase staining in CDCP1‐depleted lung cancer cells after treatment with the indicated dose of 8PN for 6 days. Scale bar, 40 μm. Statistical analyses were determined by the Student's *t*‐test. Data are shown as mean ± standard deviation. Experiments were performed in three replicates. *P < 0.05, **P < 0.01.

Next, we investigated 8PN's effects on cell cycling in lung cancer cells. These experiments showed that 8PN significantly increased the G0/G1 fraction of cells, and decreased the S phase fraction (Fig. S3A). The cell cycle assay also showed that the percentage of cells in G2/M phase declined after the 48 h 8PN treatment of TC1 and H1650 cells but not Bm7 cells. By detecting G1/S‐transition modulators upon 8PN exposure for 48 h, phosphorylated RB and cyclin E2 were significantly suppressed in Bm7 and H1650 cells (Fig. 3A). 8PN downregulated cyclin E1 in H1650 but not in Bm7 (Fig. 3A). Two cyclin‐dependent inhibitors (p27 and p21) were undetectable in Bm7 cells and showed the opposite trend in 8PN‐treated H1650 cells (Fig. 3A). This suggests that 8PN arrests lung cancer cells generally at G1/S phase, probably through reducing the RB phosphorylation and cyclin E2.

Cellular senescence prevents cell proliferation and is characterized by a stable cell cycle arrest [15]. We further investigated whether 8PN can promote cellular senescence in cancer cells by detecting β‐galactosidase in the panel of lung cancer cells. Compared with untreated cell lines, the percentage of senescent cells under 20–25 μm 8PN treatment was significantly higher: 7.8 times higher in Bm7 cells, 2.6 times higher in H1650 cells, and 5.3 times higher in TC1 cells (Fig. 3B; Fig. S3B). To verify this phenomenon is due to CDCP1‐targeted effects, we detected the senescence assays in control and CDCP1 knockdown cells. As shown in Fig. 3C, senescence cells accumulated when lung cancer cells were treated with 8PN but a slight reduction in CDCP1 knockdown cells with 8PN treatment. Together, these data reflect that 8PN triggers cell accumulation at the G0/G1 phase of the cell cycle and promotes cell senescence.

### 3.4 Combination of 8PN and EGFR TKIs synergistically reduces cell viability of EGFR mutant lung cancer cells

**Fig. 4:**
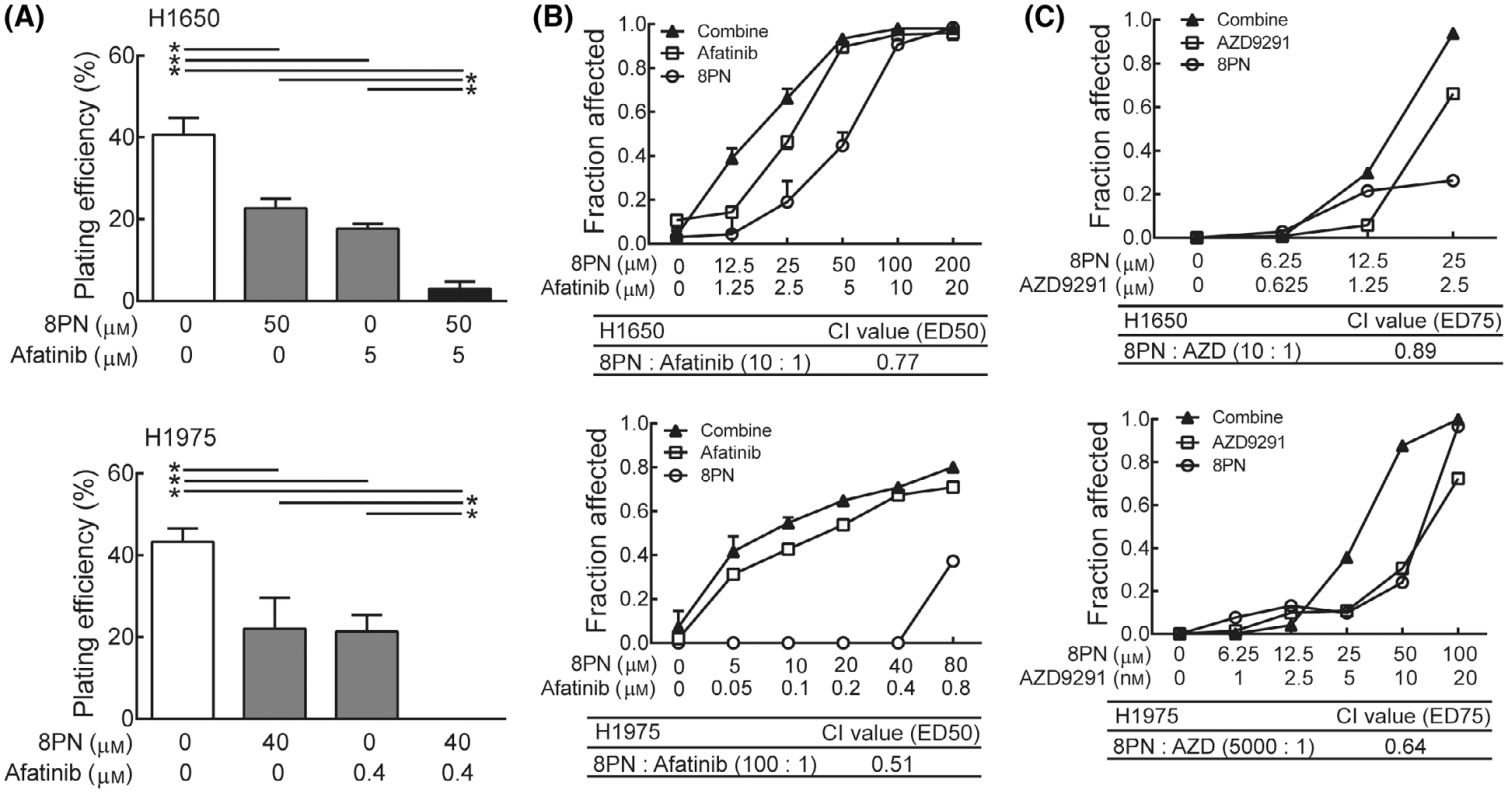
Synergistic inhibition of EGFR mutational lung cancer progression by 8PN and EGFR TKIs. (A) Combination treatment with 8PN and afatinib in H1650 and H1975 cells for 7 days. The cytotoxic effects were examined by colony formation assays (*n* = 3). Statistical analyses were determined by the Student's *t*‐test. (B) Lung cancer cells were treated with 8PN and afatinib for 72 h. Cell viability was analyzed using colony formation assays. Dose‐dependent cytotoxic fraction was plotted (top); CI at 50% inhibition (ED_50_) was calculated (bottom). (C) Cytotoxic effects were assessed by the colony formation ability of lung cancer cells upon 8PN and/or AZD exposure for 7 days. Dose‐dependent cytotoxic fraction was plotted (top); CI at 75% inhibition (ED_75_) was calculated (bottom). Data are shown as mean ± standard deviation. Experiments were performed in three replicate wells. *P < 0.05.

Next, we investigated whether 8PN‐mediated CDCP1 reduction can increase the sensitivity to EGFR TKI in EGFR TKI‐resistant lung cancer cells, including H1650 (EGFR exon 19 deletion and PTEN mutant) and H1975 (EGFR L858R) cells. Cytotoxic effects of the drug combination of 8PN and afatinib were greater compared with single drug treatment by colony formation assays (Fig. 4A). Moreover, measured CI values of 0.77 and 0.51 in H1650 and H1975 cells, respectively, demonstrated moderate synergistic effects for the two‐drug combination (Fig. 4B). Concurrently, the combination treatment caused a significant decline in cell migration, compared with the single drug treatments of 8PN and afatinib (Fig. S4). Lung cancer cells harboring an EGFR T790M mutation, such as H1975, are specifically targeted by the third‐generation EGFR TKI AZD. Consistently, the combination of 8PN and AZD led to a synergistic therapeutic effect in H1650 (CI value = 0.89) and H1975 cells (CI value = 0.64) (Fig. 4C). Taken together, 8PN shows a synergistic anticancer effect with EGFR TKIs in lung cancer treatment.

### 3.5 8PN potentiates the inhibitory effects on cell growth of afatinib and AZD by inducing apoptotic signaling

**Fig. 5:**
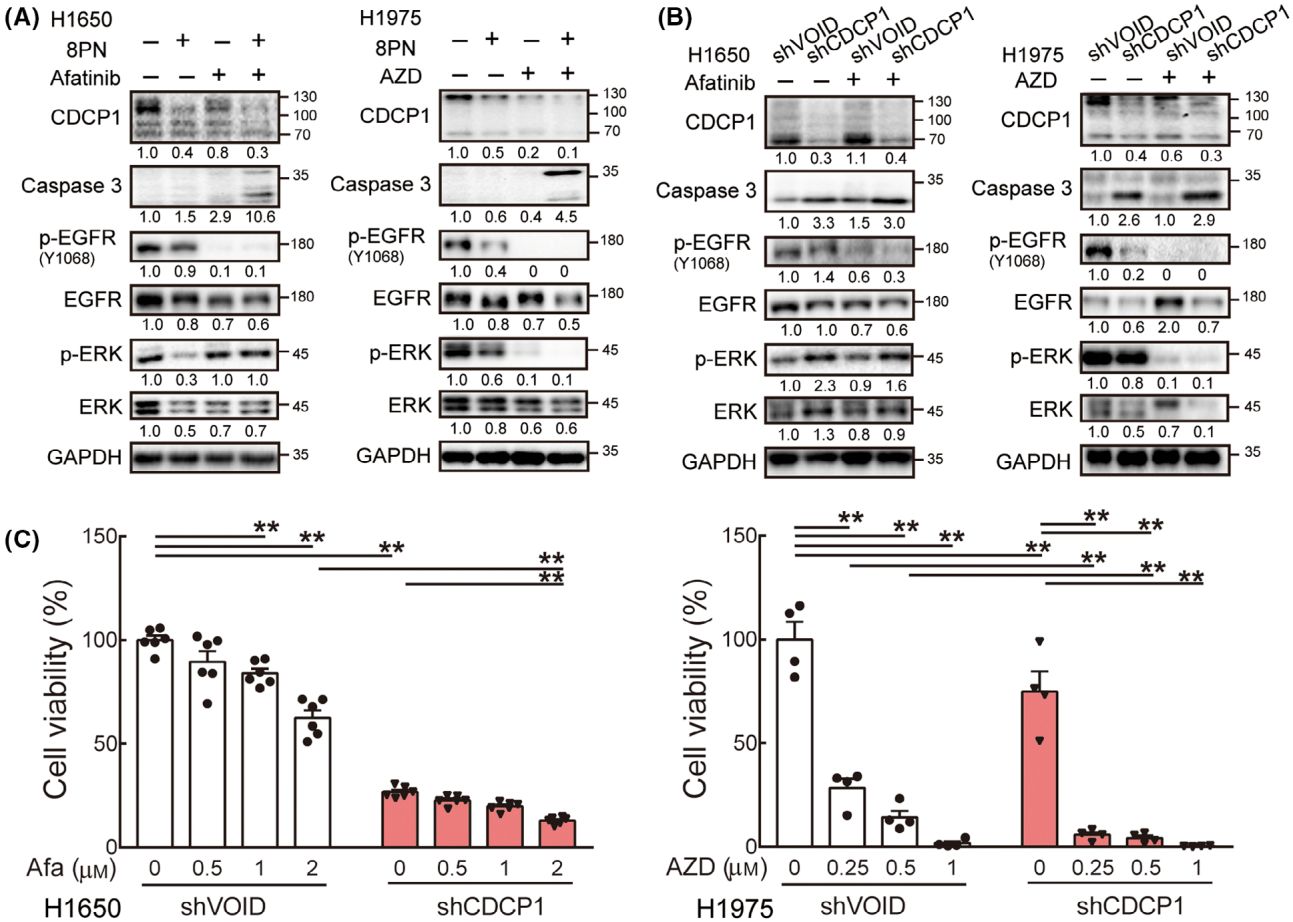
Loss of CDCP1 in lung cancer cells significantly increases lung cancer cell death. (A) Levels of CDCP1, caspase 3, phospho‐EGFR, and phospho‐ERK were detected in H1650 cells treated with 50 μm 8PN and/or 2.5 μm afatinib (left) and H1975 cells treated with 40 μm 8PN and/or 1 μm AZD (right). Experiments were repeated in three times. (B) Levels of indicated proteins were detected in control (shVOID) and CDCP1 knockdown (shCDCP1) H1650 (left) and H1975 (right) cells treated with 1 μm afatinib or 10 μm AZD. Experiments were repeated in three times. (C) H1650 (*n* = 6) and H1975 cells (*n* = 4) were treated with EGFR TKI for 3 and 9 days, respectively. The cell viability was assessed by colony formation assays. Statistical analyses were determined by the Student's *t*‐test. Data are shown as mean ± standard deviation. **P < 0.01.

To further explore the molecular mechanism underlying the synergistic effects of 8PN and afatinib or AZD in lung cancer treatment, we investigated multiple signaling pathways of cell survival in lung cancer cells. 8PN treatment suppressed CDCP1 expression, and EGFR downstream ERK phosphorylation in H1650 and H1975 cells (Fig. 5A). As expected, AZD strongly inhibited the phospho‐EGFR, and phospho‐ERK in H1975 cells, whereas afatinib blocked the EGFR phosphorylation without reducing phospho‐ERK proteins in H1650 cells (Fig. 5A). Consistent with the cytotoxicity assays, we found the combination of 8PN and AZD or afatinib increased the levels of cleaved caspase 3, a marker of apoptosis, compared with single drug treatment in H1650 and H1975 cells (Fig. 5A).

Next, we examined whether genetic reduction in CDCP1 can enhance the efficacy of TKI treatment similar to the observed effects of 8PN treatment. We knocked down CDCP1 using lentiviral shRNA in lung cancer cells. As displayed in Fig. 5B, CDCP1 silencing increased cleaved caspase 3 protein levels, but the EGFR downstream signaling was not exactly like the 8PN treatment, probably due to the cell response to long‐term CDCP1 reduction under selective pressure. Notably, treatment of CDCP1 knockdown cells with EGFR TKIs showed an increase in cleaved caspase 3 protein. Moreover, the knockdown of CDCP1 in lung cancer cells significantly lowered the cell viability upon EGFR TKI treatment compared with TKI treatment alone (Fig. 5C). These results indicate that 8PN exerts synergistic cytotoxic effects with EGFR TKIs to induce cell apoptosis, at least partly through inhibition of CDCP1.

### 3.6 A combination of 8PN and EGFR TKIs enhances antitumor effects and promotes tumor necrosis *in vivo*

**Fig. 6:**
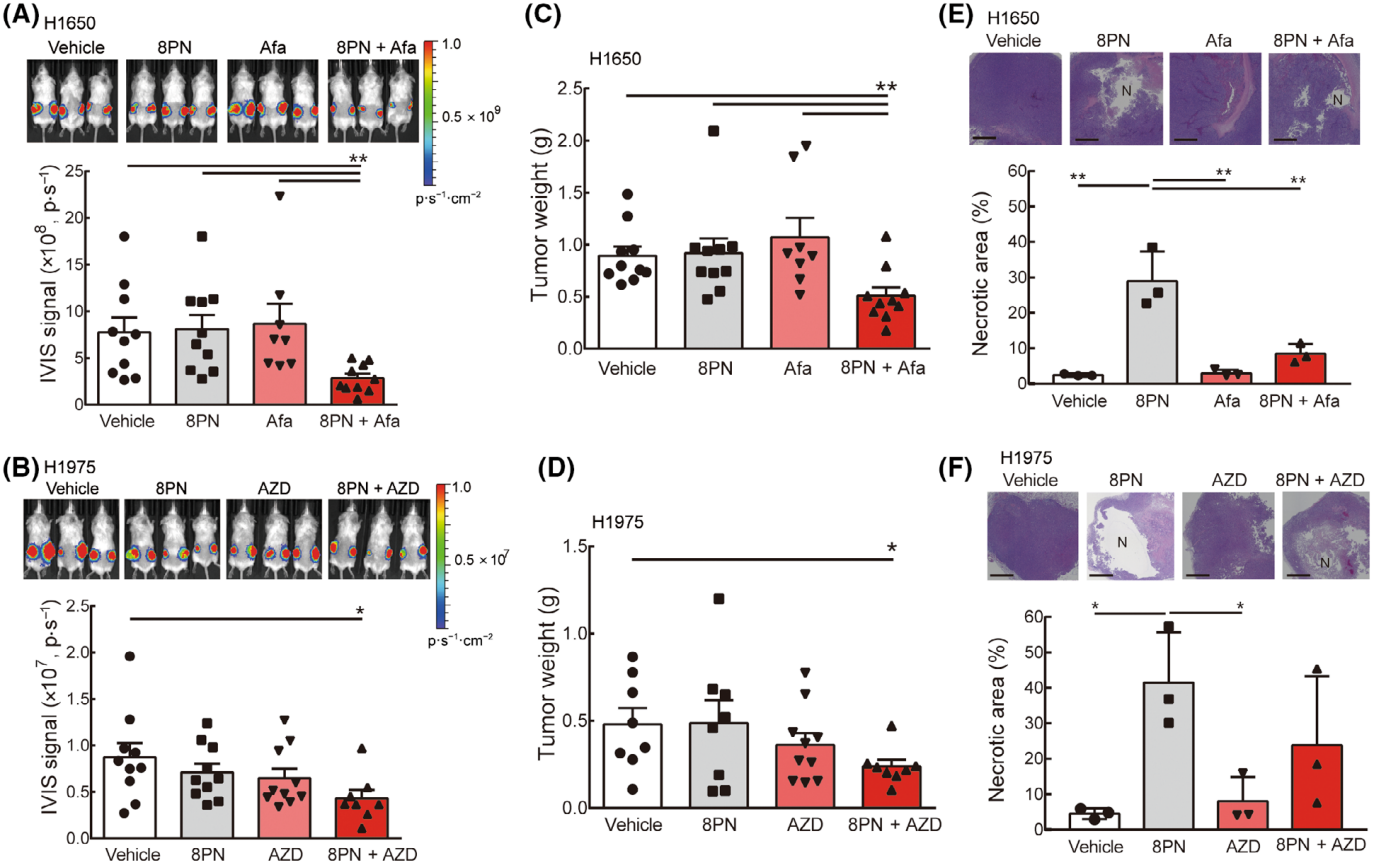
8‐Isopentenylnaringenin exerts synergistic effects with EGFR TKIs in lung cancer progression by promoting tumor necrosis. (A) Upper row: representative IVIS images of the subcutaneous H1650 tumor‐bearing mice with vehicle (*n* = 10), 10 mg·kg^−1^ 8PN (*n* = 10), 5 mg·kg^−1^ afatinib (afa; *n* = 10), or the combination (8PN + Afa; *n* = 10) on day 22. Lower row: quantification of the fluorescence signals in mice. (B) Upper row: representative bioluminescent images of H1975‐bearing mice treated with vehicle (*n* = 10), 10 mg·kg^−1^ 8PN (*n* = 10), 0.1 mg·kg^−1^ AZD (*n* = 10), or the combination (8PN + AZD; *n* = 10) on day 79. Lower row: quantification of the fluorescence signals in mice. (C, D) Quantification of excised tumor weights at the endpoint of the experiments. (E, F) Upper row: representative histological analyses of H&E stained tumor sections bearing central tumor necrosis (denoted by N). The percentage of necrotic areas was quantified (*n* = 3 mice/group). Scale bar, 1 mm. Statistical analyses were determined by the Student's *t*‐test. Data are shown as mean ± standard deviation. *P < 0.05, **P < 0.01.

Next, we assessed the synergistic effects of 8PN and EGFR TKIs *in vivo*. We established lung tumor xenograft models by subcutaneous injection of luciferase‐expressing H1650 and H1975 cells on two sides of 6–8‐week‐old SCID mice. The SCID mice were randomly divided into four groups and received daily administration of 8PN and/or EGFR TKIs. We monitored the tumor growth and determined antitumor activity by detecting fluorescence signals from the tumor cells using an IVIS imaging system. Monotherapy of 8PN and EGFR TKIs showed similar fluorescence signals to vehicle control (Fig. 6A,B). By contrast, the combination of 8PN and EGFR TKIs efficiently reduced fluorescence intensity in subcutaneous H1650 and H1975 tumors (Fig. 6A,B). Mice were sacrificed after treatment, and harvested tumors showed a significant decrease in tumor weights in the co‐treatment groups (Fig. 6C,D). Importantly, no remarkable changes in body weight of any of the treated mice were observed, compared with the vehicle control mice (Fig. S5A,B), demonstrating the negligible toxic impact of combination treatment 8PN and EGFR TKIs *in vivo*. Notably, in the histopathological analysis of tumor specimens, the largest necrosis area was detected on 8PN‐treated mice (Fig. 6E,F). Combined treatment displayed a smaller necrotic area compared with the 8PN group, whereas monotherapy of EGFR TKIs and vehicle control of tumors did not develop detectable necrosis (Fig. 6E,F). Taken together, 8PN synergizes with the therapeutic effects of EGFR TKI in mice bearing lung tumors.

### 3.7 Neutrophils enhance 8PN‐induced lung cancer cell necrosis

**Fig. 7:**
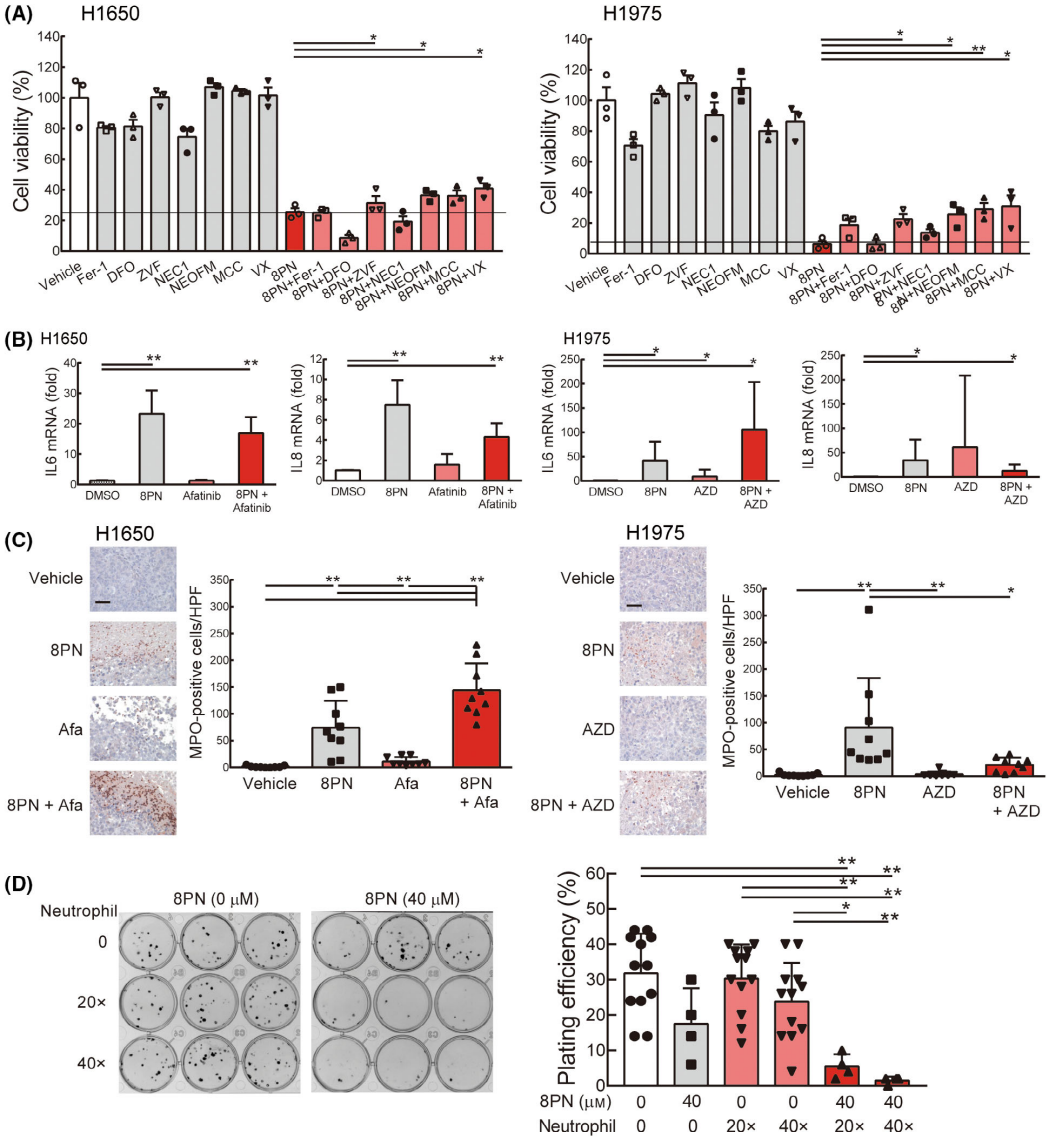
Neutrophils facilitate 8PN‐induced necrosis in lung cancer. (A) Cell viability after 48 h of 8PN treatment with vehicle, 1 μm ferrostatin (Fer‐1), 10 μm deferoxamine (DFO), 10 μm Z‐VAD‐FMK (ZVF), 50 μm necrosulfonamide (NEOFM), 2 μm MCC‐950 (MCC), or 5 μm VX765 (VX) in H1650 and H1975 cells. Assays were performed in triplicate. (B) qRT‐PCR analysis of IL6 and IL8 mRNA in H1650 and H1975 lung cancer cell lines. Experiments were repeated in three times. (C) MPO immunohistochemical staining revealed neutrophil activation. Representative MPO‐stained sections from H1650 or H1975 xenograft tumors are shown, and the corresponding MPO‐positive cells per high‐power field (HPF) were quantified (*n* = 3 mice/group; 3 random fields/mouse). Scale bars, 25 μm. (D) 8PN‐pretreated human neutrophils were co‐cultured with 8PN‐treated H1650 cells for 5 days. Cell viability was determined by the colony formation ability of H1650 cells cultured with vehicle (*n* = 12), 40 μm 8PN only (*n* = 4), human neutrophils only (*n* = 12), or human neutrophils with 8PN (*n* = 4). Representative images of colony formation assay (left panel) and the quantification of plating efficiency (right panel) are shown. Statistical analyses were determined by the Student's *t*‐test. Data are shown as mean ± standard deviation. *P < 0.05, **P < 0.01.

To understand how can 8PN cause tumor necrosis leading to tumor death, lung cancer cells were co‐treated with 8PN and a variety of small molecule inhibitors against different cell death types, including ferrostatin‐1 (ferroptosis inhibitor), deferoxamine (ferroptosis inhibitor), z‐VAD‐FMK (pan‐caspase apoptosis inhibitor), necrostatin‐1 (necroptosis inhibitor), necrosulfonamide (necroptosis inhibitor), MCC‐950 (pyroptosis inhibitor), and VX765 (pyroptosis inhibitor). These small molecule inhibitors, when used alone, had low cytotoxic effects and exhibited 80% or greater cell viability compared with vehicle controls (Fig. 7A). In comparison, low viability (10–40%) was observed when H1650 and H1975 cells were exposed to these compounds in combination with 8PN. Notably, z‐VAD‐FMK, necrosulfonamide, and VX765 significantly increased cell viability in both lung cancer cell lines compared with 8PN treatment alone, but the cell survival did not reach the level of major recovery (Fig. 7A). Consistent results were observed that the sub‐G1 portions were increased when H1650 cells were treated with 8PN for 24 h (Fig. S6A). By contrast, the sub‐G1 percentage remained at a low level in CDCP1 knockdown cells with vehicle or 8PN treatment (Fig. S6A). This suggests that 8PN targets CDCP1 and acts via multiple alternative necrotic lung cancer cell death pathways, including apoptosis, necroptosis, and pyroptosis. However, the effects of 8PN‐mediated cell death pathways were not potent enough to explain the strong necrosis observed in xenograft tumor models.

Next, we asked whether 8PN can regulate the cytokine profile and alter the tumor microenvironment for promoting tumor necrosis. Because previous evidence showed that cytokine IL6 and IL8 secretion promoted necrosis in glioblastoma [16], we examined these two cytokines in lung cancer cells treated with 8PN or/and EGFR TKIs. Indeed, IL6 and IL8 mRNA levels were dramatically increased on 8PN treatment, either alone or in combination with EGFR TKIs, in H1650 and H1975 cells (Fig. 7B). We also found that EGFR TKIs enhanced IL6 mRNA expression in CDCP1 knockdown lung cancer cells (Fig. S6B). Previous studies reported that IL6 and IL8 were associated with neutrophil recruitment, which can be verified by a human‐activated neutrophil marker, MPO [16, 17]. MPO‐expressing cells were abundant in 8PN‐triggered necrotic areas under high‐power magnification, yet were rarely found in tumors of mice receiving EGFR TKI monotherapy or vehicle (Fig. 7C; Fig. S6C). We further confirmed that 8PN increased intracellular ROS production on H1650 and neutrophils independently (Fig. S6D), indicating that 8PN activates neutrophils and increases the ROS levels in cancer cells.

Next, to assess whether 8PN can activate neutrophils to induce tumor necrosis, lung cancer cells, and neutrophils were pretreated with 8PN independently, and then mixed together to co‐culture with 8PN for further assessing cancer cell viability using colony formation assays. Based on the number of infiltrating neutrophils surrounding the tumor cells from the xenograft tumor specimens, we modeled the tumor environment with ratios of 20 : 1 and 40 : 1 neutrophils to lung cancer cells in the cell‐based co‐culture system. We found that a high number of neutrophils (40‐fold) slightly reduced colony formation in the absence of any other treatments, as did exposure to 8PN alone (Fig. 7D). However, a stronger growth inhibitory effect on H1650 cells was detected upon co‐treatment with 8PN and neutrophils (Fig. 7D). Anti‐IL6 receptor antibody (Tocilizumab) did not affect the recovery of colony formation in H1650 cells upon co‐treatment with 8PN and neutrophils (Fig. S6E). To investigate the role of ROS after 8PN‐treated neutrophils in lung cancer cells, we showed that antioxidant NAC reduced the 8PN‐mediated intracellular ROS in neutrophils, but it cannot change the ROS levels of cancer cells by detecting DCFH‐DA fluorescence with flow cytometry (Fig. S6F). Moreover, NAC had no effect to rescue the growth inhibitory effect on H1650 cells upon co‐treatment with 8PN and neutrophils (Fig. S6G). It suggested that 8PN‐mediated neutrophils for killing cancer cells were complicated and ROS may contribute little. Overall, these results indicated that 8PN regulates the cell death pathway by activating neutrophils, and promotes intrinsic necrosis that synergizes with the anticancer effects of EGFR TKIs.

## 4 Discussion

CUB domain‐containing protein 1 plays a variety of promoting roles in EGFR‐driven lung cancer. Blocking CDCP1 using antibodies or small molecule inhibitors has been shown to effectively attenuate tumor development [18, 19]. For example, glycoconjugated palladium complex (Pd‐Oqn), a small compound, was shown to reduce tumor growth and metastasis by blocking the interaction of phosphorylated CDCP1 and PKC γ [19]. High binding affinity of antibodies against the cleavage sites of CDCP1 was demonstrated to decelerate CDCP1‐driven tumor development [18]. In the present study, we identified 8PN, a natural compound, as a CDCP1 inhibitor to suppress lung cancer progression.

8‐Isopentenylnaringenin, a prenylated flavonoid, is a natural hop‐derived compound used in the brewing industry [20]. 8PN exhibits antiproliferative properties in human colon, prostate, and ovarian cancer cells [21, 22]. Moreover, 8PN induces cell cycle arrest by attenuating cell growth via the downregulation of cyclin D in MCF7 breast cancer cells [23]. However, no studies have focused on 8PN effectiveness in lung cancer, based on our best knowledge. In line with previous studies [22, 23], we found that 8PN triggered lung cancer cell accumulation at G0/G1 and elevated the proportion of senescent cells. However, apoptotic induction in 8PN treatment was not detected by measuring the abundance of cleaved caspase 3, which is consistent with previous observations [24, 25]. Surprisingly, the combination of 8PN and EGFR TKIs induced apoptosis, as indicated by cleavage of caspase 3, revealing that the combination treatment could trigger programmed cell death in lung cancer cells. Moreover, we found that 8PN induced cell senescence in lung cancer cells, consistent with the report that overexpression of CDCP1 bypasses senescence to increase prostate cancer cell proliferation [26].

We found that 8PN functioned as a CDCP1 inhibitor and reduced the EGFR downstream signaling. However, in CDCP1 knockdown cells, we observed that p‐ERK proteins were elevated in H1650 and H1975, respectively, which differed from the 8PN treatment. The paradoxical results between 8PN treatment and CDCP1 knockdown cells are unclear. Although growth factor signaling can enhance the ERK pathway activation by PDGF and RAS activation for inducing CDCP1 expression [4], whether CDCP1 has a feedback regulation for influencing ERK signaling is still unknown. We observed that CDCP1 knockdown cells showed a high level of caspase 3 and these cells disappeared in the pooled cell population after several passages. It raised the possibility that alternative signaling pathway activation had occurred to sustain cell viability in CDCP1 knockdown cells. Another probability is that 8PN has multiple targets in addition to CDCP1 that lead to reducing EGFR downstream signaling. Our results reveal that 8PN also reduced the levels of phosphorylated RB and cyclin E2 in lung cancer cells (Fig. 3A), which leads to cell cycle arrest and inhibition of lung cancer cell viability. It suggests that 8PN has multiple targets in addition to CDCP1, but this needs further exploration.

As a phytoestrogen, 8PN has estrogenic effects, including stimulating the mammalian estrogen receptor (ER), with multiple physiological manifestations [23, 27]. Several studies also found that ER associates with EGFR, providing an alternative signal for clinical outcome optimizations [28, 29]. High levels of ER or EGFR showed a significant correlation with poor overall survival in NSCLC patients, suggesting that the ER‐triggered EGFR signaling axis promotes tumor progression [30, 31]. Our results clearly demonstrate that 8PN reduced phosphorylated EGFR protein levels. Importantly, EGFR was inactivated in response to the combination of 8PN and EGFR TKIs. These observations suggest that 8PN attenuates lung cancer progression via an EGFR‐dependent signaling pathway. By comparing the effects of CDCP1 knockdown and EGFR TKI, we found that CDCP1 is crucial for H1650 cell viability, whereas EGFR is for H1975 cells (Fig. 5C). H1650 (EGFR exon 19 deletion and PTEN mutant) cells are resistant to EGFR TKIs due to ERK activation, whereas H1975 (EGFR L858R) cells are sensitive to AZD. We found that the combination of 8PN and EGFR TKIs showed a slight synergism (CI values: 0.77 and 0.89) in H1650, whereas it showed a moderate synergism (CI values: 0.51 and 0.64) in H1975. Although the combination treatment reduced the EGFR phosphorylation in the two cell lines, p‐ERK levels remained high in H1650 compared with H1975. It suggests that the influence of EGFR‐dependent signaling pathways is related to the response of combined therapy. However, the association of 8PN‐dependent EGFR signaling and ER in lung cancer has not yet been clarified in this study. Collectively, our results reflect that the combination of 8PN and EGFR TKI exerts a strong inhibitory effect on lung cancer cells and might be an effective chemotherapy for lung cancer patients.

Cell death is an essential physiological process in multicellular organisms, and has recently been classified into several types corresponding to the cellular morphology, including necroptosis, ferroptosis, and pyroptosis [32]. Here, our animal models have contributed to our understanding of the synergistic effect of 8PN and TKIs, yielding to cancer cell death. Interestingly, the necrotic area in mice treated with the combination of 8PN and TKI was lower than that in mice treated with 8PN alone. Emerging evidence reveals that tumor necrosis is a complex consequence of metabolic stress and inflammation [33]. Neutrophil recruitment plays a key role in amplifying tumor necrosis and our study also supports this point. So far, the relationship between tumor necrosis and tumor progression is still controversial and may be dependent on the tumor development stages. Our results revealed that a combination of 8PN and TKI induced strong cell apoptosis compared with monotherapy, and showed smaller tumor size. Moreover, minimal cell viability was restored upon co‐treatment of 8PN and inhibitors of regulated cell death, suggesting that 8PN‐mediated cancer cell death is a combined effect, not limited to a single pathway. It implies that developing multiple cell death pathways simultaneously in a tumor environment may synergize the antitumor effects, resulting in a lower necrotic area in combination therapy compared with monotherapy. However, this needs to be further studied.

8‐Isopentenylnaringenin also stimulated cell‐mediated killing pathways by increasing IL6 and IL8 expression. IL6 mediates neutrophil mobilization from bone marrow [34], and IL8 enhances neutrophil recruitment for increased colorectal cancer cell death [35]. Notably, IL6 mRNA level was lower in H1650 cells with combination therapy of 8PN and afatinib compared with 8PN alone. Consistently, the necrotic area in the combination group was significantly decreased compared with the 8PN group in H1650 tumors. In H1975 cells, IL6 was strongly elevated in the 8PN alone and in combination therapy of 8PN and AZD, and it showed a comparable necrotic area in the H1975 tumors. However, anti‐IL6 receptor antibody did not significantly recover the cell growth ability. It suggests that IL6 is likely for neutrophil recruitment rather than activation of 8PN‐mediated neutrophil‐triggered tumor cytotoxicity. Thus, we reveal that 8PN provides multiple anticancer effects, including intrinsic apoptosis pathways in lung cancer cells, cytokine release for neutrophil attraction, and neutrophil‐mediated cytotoxicity.

## 5 Conclusions

**Fig. 8:**
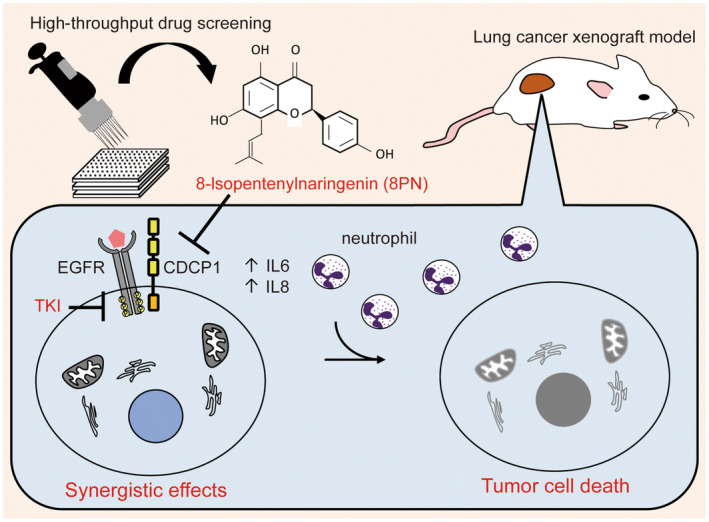
Schematic diagram showing that 8PN reduces CDCP1 protein levels and increases IL6 and IL8 expression in lung tumors to attract neutrophil infiltration. The combination of 8PN and EGFR TKI produces synergistic effects in lung cancer treatment.

In summary, 8PN, a natural compound, was identified as a CDCP1 inhibitor that can attenuate lung cancer cell malignancy. Importantly, the combination of 8PN and EGFR TKIs demonstrated synergistic anticancer effects on EGFR TKI‐resistant lung tumor xenografts. We provide clear evidence that 8PN treatment attracts neutrophils and triggers necrotic lung cancer cell death to effectively overcome EGFR TKI resistance (Fig. 8). Our results highlight that 8PN has the potential for further development as an adjuvant for improving the efficacy of EGFR TKIs in lung cancer treatment.

## Conflict of interest

The authors declare no conflict of interest.

## Author contributions

S‐CW, X‐YZ, C‐YH, C‐CLo, and T‐TK performed the experiments and analyzed the data. C‐CY, L‐CC, C‐HC, and L‐HW contributed reagents and participated in the study conception. S‐CW, C‐CLi, and J‐PL performed the neutrophil assays. S‐CW, C‐CY, and Y‐PS wrote the manuscript and revised it critically for important intellectual content. All authors read and approved the final manuscript.

### Peer Review

The peer review history for this article is available at https://www.webofscience.com/api/gateway/wos/peer‐review/10.1002/1878‐0261.13429.

## Supporting information


**Fig. S1.** 8PN increased CDCP1 protein degradation and suppressed lung cancer cell viability. (A) Relative mRNA level of CDCP1 in TC1 with 20 μM 8PN and Bm7 with 25 μM 8PN for 24 h. (B) Immunoblotting analysis of CDCP1 in Bm7 with 25 μM 8PN and H1650 with 75 μM 8PN for 24 h. Four hours before harvest, cells were treated in the presence or absence of 5 μM MG132 or 20 μM chloroquine (CQ) in Bm7 cells. 20 μM MG132 or 40 μM CQ in H1650 cells. GAPDH was the loading control. (C) 8PN dose‐dependently suppressed lung cancer cell proliferation. MTT assay was performed in Bm7, H1650, TC1, and splenocytes upon 8PN treatment for 72 h.

**Fig. S2.** CDCP1 knockdown suppresses sphere formation ability and activates caspase 3‐induced apoptosis. (A) Immunoblotting analysis of CDCP1 in control and CDCP1 knockdown Bm7 cells. Actin was the loading control (left). The number of spheres was measured after 7 days in control and CDCP1 knockdown Bm7 cells (right). Scale bar, 50 μm. Statistical analyses were determined by the Student's *t*‐test. *, *p* < 0.05. (B) Immunoblotting analysis of CDCP1 and caspase 3 in CDCP1‐overexpressed (OE) or CDCP1 knockdown H1650 with 50 μM 8PN for 24 h. GAPDH was the loading control.

**Fig. S3.** Effect of 8PN on cell cycle arrest and cell senescence. (A) Cell cycle distribution was determined by flow cytometry after 8PN treatment for 48 h of TC1 cells with 20 μM 8PN, Bm7 cells with 25 μM 8PN, and H1650 cells with 50 μM 8PN. (B) β‐Galactosidase staining in TC1 with 20 μM 8PN for 6 days. The number of senescent cells was quantified using the representative images shown in the upper panel. Scale bar, 40 μm. Statistical analyses were determined by the Student's *t*‐test. *, *p* < 0.05.

**Fig. S4.** Synergistic inhibition of lung cancer migration ability by 8PN and EGFR TKIs. Cells were treated with 8PN and/or afatinib for 17 h, and the migration distance of H1650 and H1975 lung cancer cells was detected by time‐lapse migration assays. The cumulative cell migration distance was calculated and shown. Statistical analyses were determined by the Student's *t*‐test. Data are shown as mean ± standard deviation. *, *p* < 0.05.

**Fig. S5.** No significant changes in body weights in treatment groups of tumor‐bearing mice. Average body weights of SCID mice bearing lung tumors at the endpoint of the experiments (n = 5 per group). Data are shown as mean ± standard deviation.

**Fig. S6.** 8PN triggers necrosis by generating ROS production. (A) Cell cycle distribution was determined by flow cytometry after 8PN treatment for 24 h of CDCP1‐depleted H1650 cells with 50 μM 8PN. (B) qRT‐PCR analysis of IL6 and IL8 RNAs in control (shVOID) and CDCP1 knockdown (shCDCP1) lung cancer cells. (C) Representative MPO‐stained sections from xenograft tumors from the low‐power field. Scale bars, 200 μm. (D) Intracellular ROS productions, as indicated by dichlorodihydrofluorescein diacetate (DCFH‐DA), in human neutrophils and H1650 cells in the presence or absence of 50 μM 8PN were quantified. (E) Human neutrophils and H1650 were independently treated with 40 μM 8PN in the presence or absence of anti‐IL6 receptor antibody (tocilizumab) for 1 day. Pretreated neutrophils and H1650 cells were subsequently co‐cultured for 5 days. Colony formation ability of H1650 cells cultured with vehicle (n = 8), 40 μM 8PN only (n = 4), human neutrophils with 8PN (n = 4) or human neutrophils with 8PN, and 200 μg/mL anti‐IL6 receptor antibody (n = 4). (F) Human neutrophils and H1650 cells were pretreated with NAC for 30 min. Neutrophils were subsequently treated with 8PN for 1 h, and H1650 cells were treated with 8PN for 24 h. Intracellular levels of ROS were then measured and quantified. (G) H1650 cells were stimulated for 30 min with 5 mM NAC and co‐cultured with 50 μM 8PN or presence with neutrophils. Cell viability was measured by crystal violet assay. Statistical analyses were determined by the Student's *t*‐test. Data are shown as mean ± standard deviation. *, *p* < 0.05. **, *p* < 0.01.

## Data Availability

The data generated and/or analyzed during the current study are available from the corresponding author upon reasonable request.
