# Supplementary figures and images for: Inhibition of CDCP1 by 8‐isopentenylnaringenin synergizes with EGFR inhibitors in lung cancer treatment
Source: Mol Oncol. 2023 Apr 20;17(8):1648–65. doi: 10.1002/1878-0261.13429 (PMC10399713; doi:10.1002/1878-0261.13429)

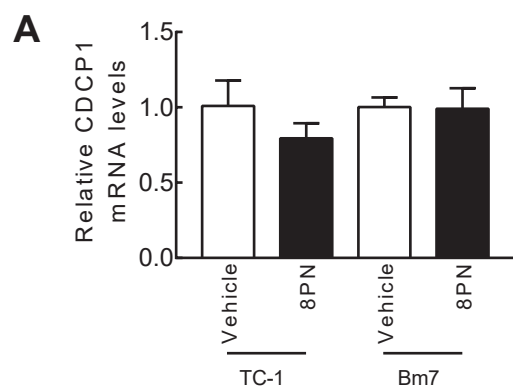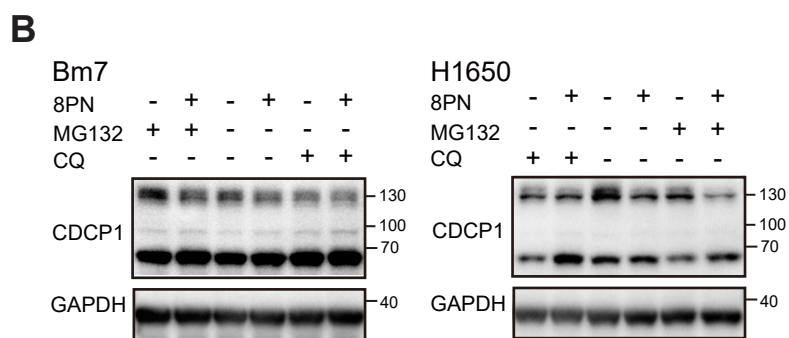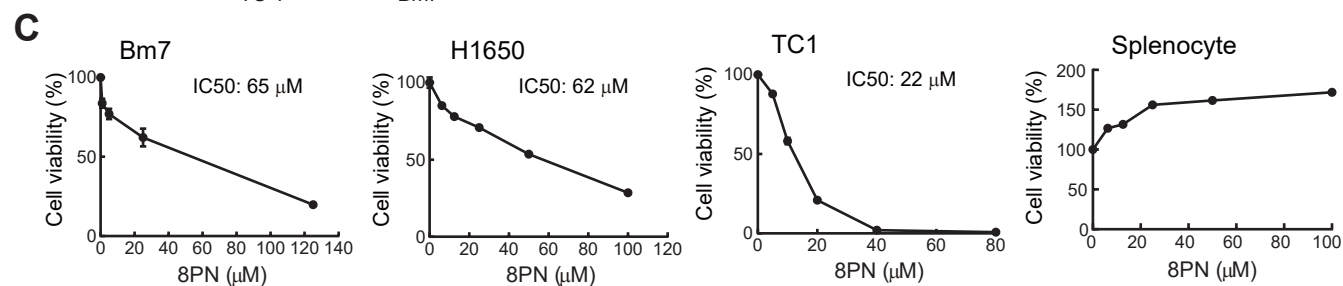

Supplement: Supplementary file 1 — Fig. S1. 8PN increased CDCP1 protein degradation and suppressed lung cancer cell viability. (A) Relative mRNA level of CDCP1 in TC1 with 20 μM 8PN and Bm7 with 25 μM 8PN for 24 h. (B) Immunoblotting analysis of CDCP1 in Bm7 with 25 μM 8PN and H1650 with 75 μM 8PN for 24 h. Four hours before harvest, cells were treated in the presence or absence of 5 μM MG132 or 20 μM chloroquine (CQ) in Bm7 cells. 20 μM MG132 or 40 μM CQ in H1650 cells. GAPDH was the loading control. (C) 8PN dose‐dependently suppressed lung cancer cell proliferation. MTT assay was performed in Bm7, H1650, TC1, and splenocytes upon 8PN treatment for 72 h. [file MOL2-17-1648-s003.pdf]

**A**

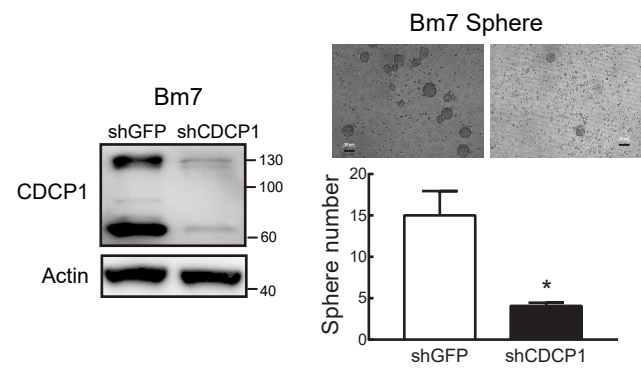

**B**

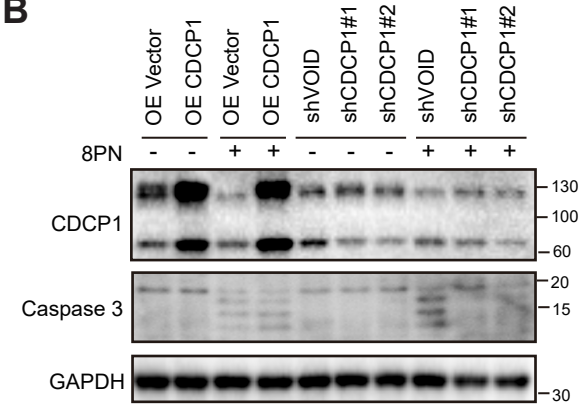

Supplement: Supplementary file 2 — Fig. S2. CDCP1 knockdown suppresses sphere formation ability and activates caspase 3‐induced apoptosis. (A) Immunoblotting analysis of CDCP1 in control and CDCP1 knockdown Bm7 cells. Actin was the loading control (left). The number of spheres was measured after 7 days in control and CDCP1 knockdown Bm7 cells (right). Scale bar, 50 μm. Statistical analyses were determined by the Student's t‐test. *, p < 0.05. (B) Immunoblotting analysis of CDCP1 and caspase 3 in CDCP1‐overexpressed (OE) or CDCP1 knockdown H1650 with 50 μM 8PN for 24 h. GAPDH was the loading control. [file MOL2-17-1648-s002.pdf]

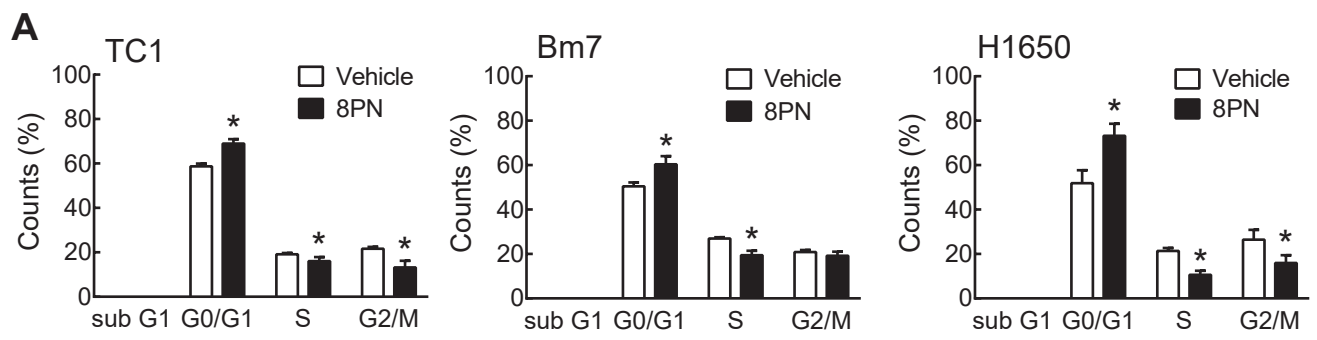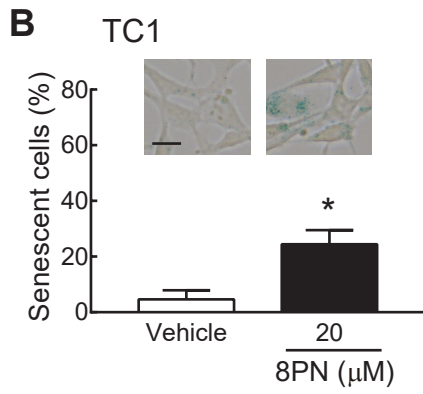

Supplement: Supplementary file 3 — Fig. S3. Effect of 8PN on cell cycle arrest and cell senescence. (A) Cell cycle distribution was determined by flow cytometry after 8PN treatment for 48 h of TC1 cells with 20 μM 8PN, Bm7 cells with 25 μM 8PN, and H1650 cells with 50 μM 8PN. (B) β‐Galactosidase staining in TC1 with 20 μM 8PN for 6 days. The number of senescent cells was quantified using the representative images shown in the upper panel. Scale bar, 40 μm. Statistical analyses were determined by the Student's t‐test. *, p < 0.05. [file MOL2-17-1648-s001.pdf]

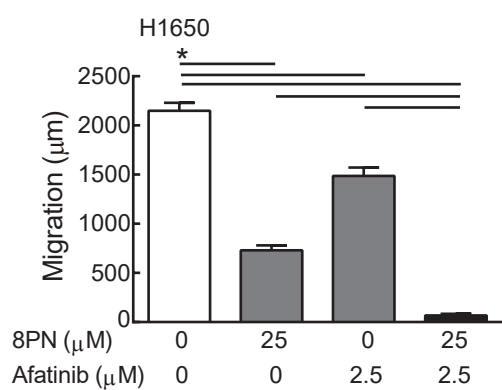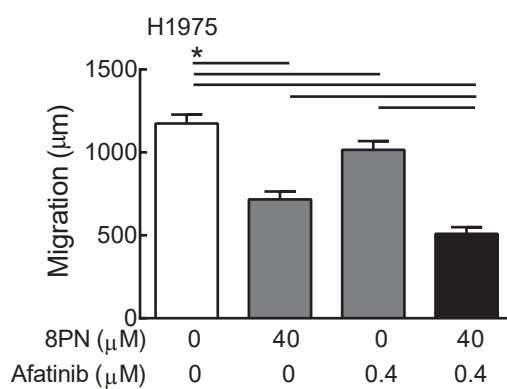

Supplement: Supplementary file 4 — Fig. S4. Synergistic inhibition of lung cancer migration ability by 8PN and EGFR TKIs. Cells were treated with 8PN and/or afatinib for 17 h, and the migration distance of H1650 and H1975 lung cancer cells was detected by time‐lapse migration assays. The cumulative cell migration distance was calculated and shown. Statistical analyses were determined by the Student's t‐test. Data are shown as mean ± standard deviation. *, p < 0.05. [file MOL2-17-1648-s005.pdf]

**A** H1650 tumor mouse model

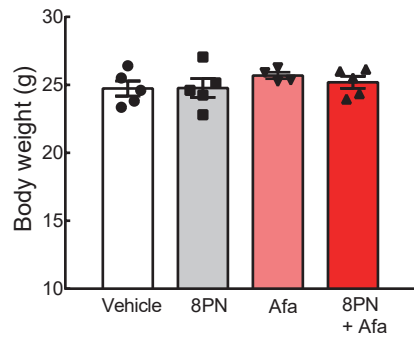

**B** H1975 tumor mouse model

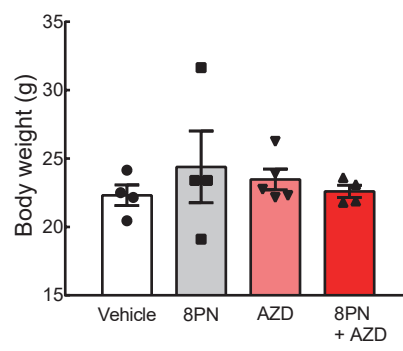

Supplement: Supplementary file 5 — Fig. S5. No significant changes in body weights in treatment groups of tumor‐bearing mice. Average body weights of SCID mice bearing lung tumors at the endpoint of the experiments (n = 5 per group). Data are shown as mean ± standard deviation. [file MOL2-17-1648-s004.pdf]

**A**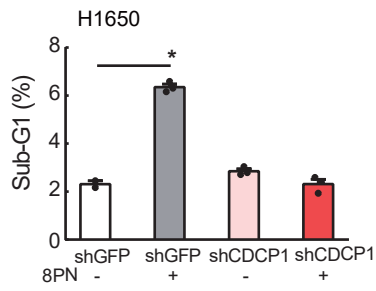**C**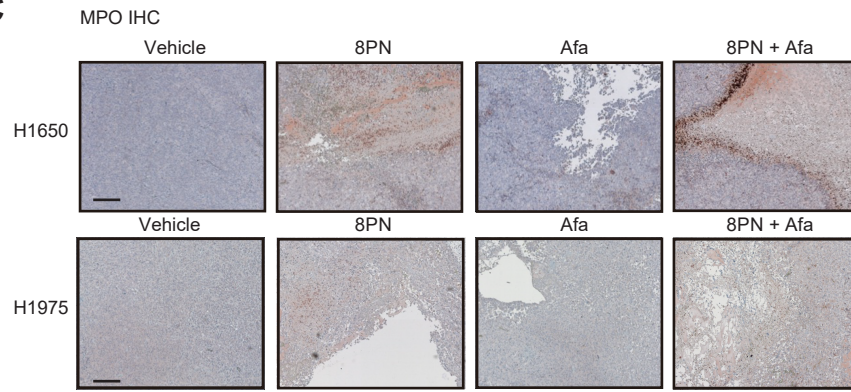**B**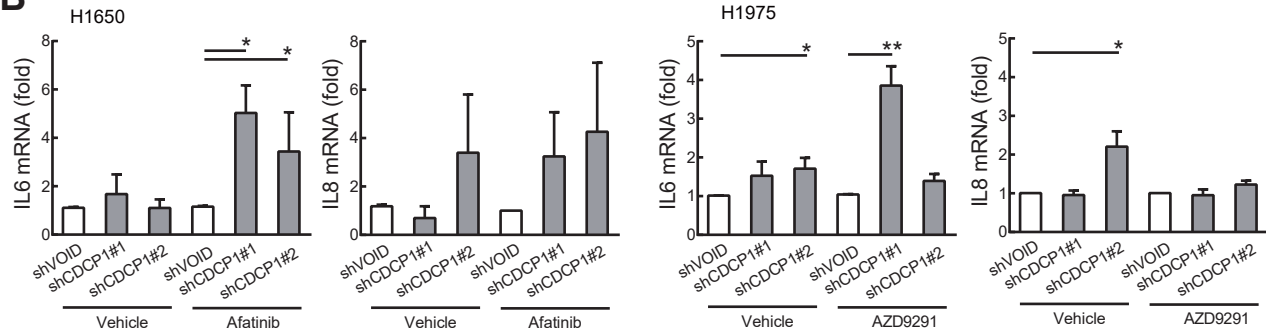**D**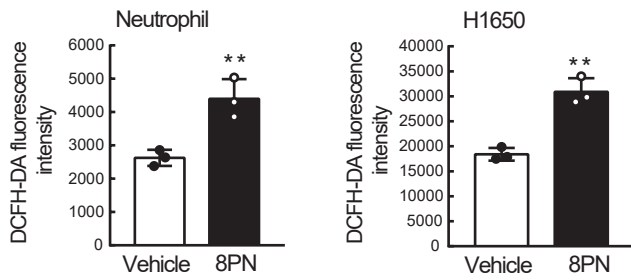**E**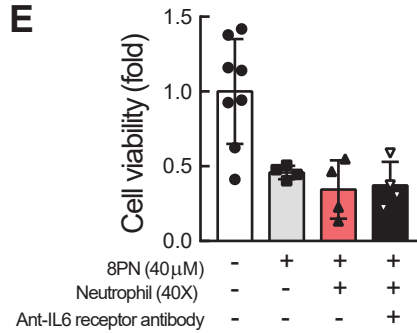**F**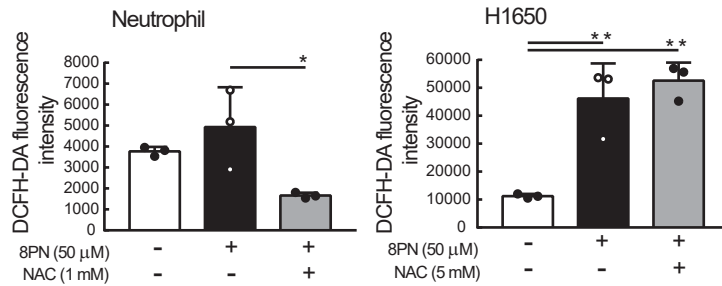**G**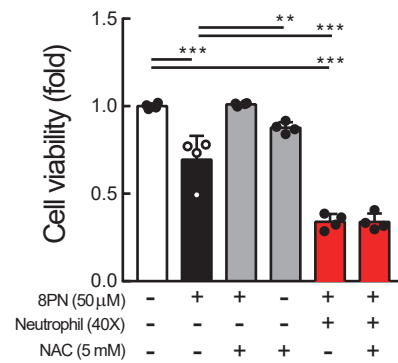

Supplement: Supplementary file 6 — Fig. S6. 8PN triggers necrosis by generating ROS production. (A) Cell cycle distribution was determined by flow cytometry after 8PN treatment for 24 h of CDCP1‐depleted H1650 cells with 50 μM 8PN. (B) qRT‐PCR analysis of IL6 and IL8 RNAs in control (shVOID) and CDCP1 knockdown (shCDCP1) lung cancer cells. (C) Representative MPO‐stained sections from xenograft tumors from the low‐power field. Scale bars, 200 μm. (D) Intracellular ROS productions, as indicated by dichlorodihydrofluorescein diacetate (DCFH‐DA), in human neutrophils and H1650 cells in the presence or absence of 50 μM 8PN were quantified. (E) Human neutrophils and H1650 were independently treated with 40 μM 8PN in the presence or absence of anti‐IL6 receptor antibody (tocilizumab) for 1 day. Pretreated neutrophils and H1650 cells were subsequently co‐cultured for 5 days. Colony formation ability of H1650 cells cultured with vehicle (n = 8), 40 μM 8PN only (n = 4), human neutrophils with 8PN (n = 4) or human neutrophils with 8PN, and 200 μg/mL anti‐IL6 receptor antibody (n = 4). (F) Human neutrophils and H1650 cells were pretreated with NAC for 30 min. Neutrophils were subsequently treated with 8PN for 1 h, and H1650 cells were treated with 8PN for 24 h. Intracellular levels of ROS were then measured and quantified. (G) H1650 cells were stimulated for 30 min with 5 mM NAC and co‐cultured with 50 μM 8PN or presence with neutrophils. Cell viability was measured by crystal violet assay. Statistical analyses were determined by the Student's t‐test. Data are shown as mean ± standard deviation. *, p < 0.05. **, p < 0.01. [file MOL2-17-1648-s006.pdf]
